# Supplementary material for: Chloroplast acquisition without the gene transfer in kleptoplastic sea slugs, Plakobranchus ocellatus
Source: eLife. 2021 Apr 27;10:e60176. doi: 10.7554/eLife.60176 (PMC8079154; doi:10.7554/eLife.60176)
Supplement: Figure 5—source data 2. — This zip archive contains source files of KRM gene searching based on the comparative genomic (orthologous) analysis. [file elife-60176-fig5-data2.zip › Figure_5_source_data_2/1_species_for_comparative_genomics.docx]

Applied species for the comparative genomic analysis

| Abbreviation name in figures | Pictogram | Scientific name | Data source |
| --- | --- | --- | --- |
| Mmu | 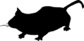 | *Mus musculus* | Uniprot; 10090 ("Reviewed" genes only) |
| Hsa | 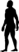 | *Homo sapiens* | Uniprot; 9606 ("Reviewed" genes only) |
| Dre | 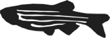 | *Danio rerio* | Uniprot;7739 |
| Bfl | 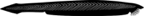 | *Branchiostoma floridae* | Uniprot;7955 |
| Dme | 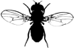 | *Drosophila melanogaster* | Uniprot; 7227 |
| Bmo | 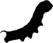 | *Bombyx mori* | Uniprot; 7091 |
| Api | 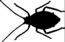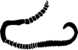 | Acyrthosiphon pisum | Uniprot; 7029 |
| Cte |  | *Capitella teleta* | Uniprot;283909 |
| Lan | 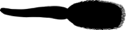 | *Lingula anatina* (synonym *unguis*) | Uniprot; 7574 |
| Obi | 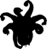 | *Octopus bimaculoides* | INSDC; PRJNA270931 |
| Cgi | 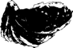 | *Crassostrea gigas (oyster_v9)* | INSDC; PRJNA276446 |
| Pfu | 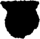 | *Pinctada fucata (v2.0)* | http://marinegenomics.oist.jp/pearl/viewer/info?project_id=36 |
| Hdi | 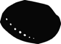 | *Haliotis discus* | <http://gigadb.org/dataset/100281> |
| Lgi | 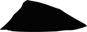 | *Lottia gigantea* | INSDC; PRJNA259762 |
| Bgl | 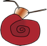 | *Biomphalaria glabrata (ASM45736v1)* | INSDC: PRJNA290623 |
| Aca | 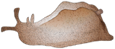 | *Aplysia californica (*AplCal3.0) | INSDC; PRJNA209509 |
| PoB | 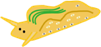 | *Plakobranchus ocellatus* type black | Present study |
| Ema | 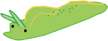 | *Elysia marginata* | Present study |
