# Supplementary figures and images for: Chloroplast acquisition without the gene transfer in kleptoplastic sea slugs, Plakobranchus ocellatus
Source: eLife. 2021 Apr 27;10:e60176. doi: 10.7554/eLife.60176 (PMC8079154; doi:10.7554/eLife.60176)

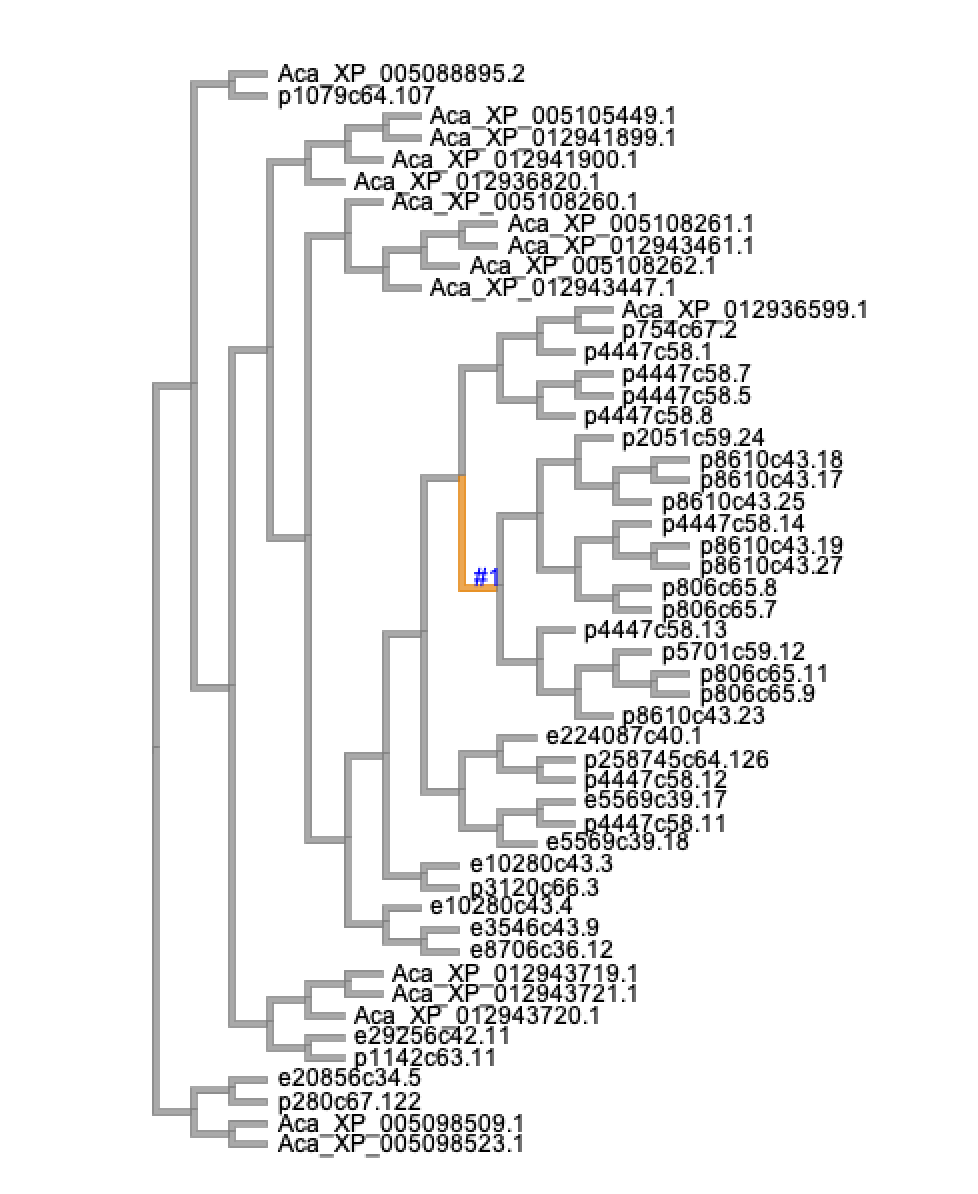

Supplement: Supplementary file 12. [file elife-60176-supp12.zip › Supplementary_file_12/OG0000446/Clade_Ia /CodeML/tree.png]
