# Supplementary material for: Chloroplast acquisition without the gene transfer in kleptoplastic sea slugs, Plakobranchus ocellatus
Source: eLife. 2021 Apr 27;10:e60176. doi: 10.7554/eLife.60176 (PMC8079154; doi:10.7554/eLife.60176)
Supplement: Supplementary file 21. [file elife-60176-supp21.zip › Supplementary_file_21/all_data_plot.pdf]

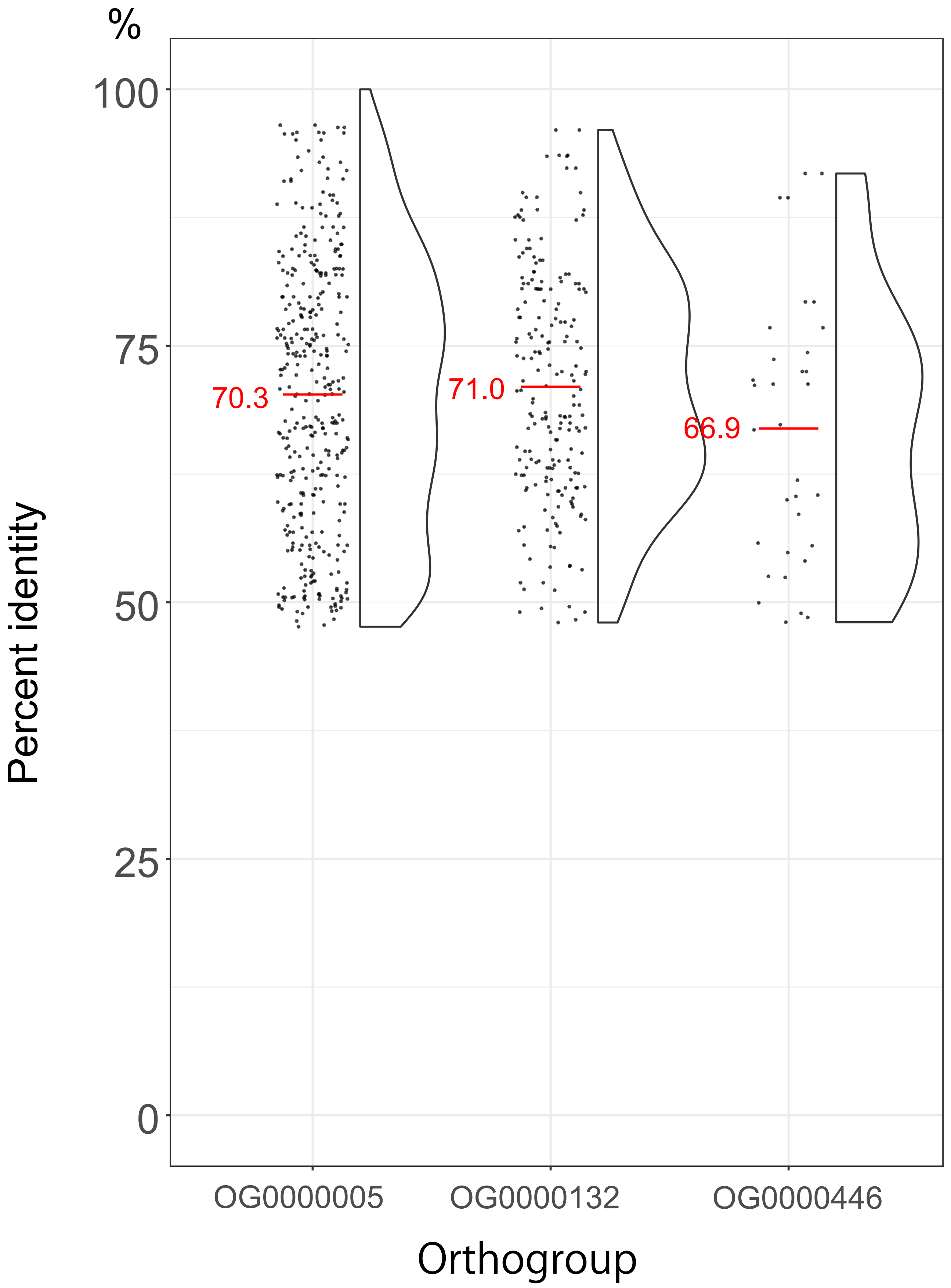

Pairwise parcent simiralry of the ortholgourp having KRM candidates.
Each dot indicates the pairwise percent similarity of sequences in each orthogroup Red line, the mean value of the similarity. The Violin plot shows the distribution of the similarity.
