# Supplementary material for: Chloroplast acquisition without the gene transfer in kleptoplastic sea slugs, Plakobranchus ocellatus
Source: eLife. 2021 Apr 27;10:e60176. doi: 10.7554/eLife.60176 (PMC8079154; doi:10.7554/eLife.60176)
Supplement: Transparent reporting form [file elife-60176-transrepform.docx]

***eLife’s* transparent reporting form**

We encourage authors to provide detailed information *within their submission* to facilitate the interpretation and replication of experiments. Authors can upload supporting documentation to indicate the use of appropriate reporting guidelines for health-related research (see [EQUATOR Network](http://www.equator-network.org/%20)), life science research (see the [BioSharing Information Resource](https://biosharing.org/)), or the [ARRIVE guidelines](http://www.plosbiology.org/article/info:doi/10.1371/journal.pbio.1000412) for reporting work involving animal research. Where applicable, authors should refer to any relevant reporting standards documents in this form.

If you have any questions, please consult our Journal Policies and/or contact us: [editorial@elifesciences.org](mailto:editorial@elifesciences.org).

**Sample-size estimation**

- You should state whether an appropriate sample size was computed when the study was being designed
- You should state the statistical method of sample size computation and any required assumptions
- If no explicit power analysis was used, you should describe how you decided what sample (replicate) size (number) to use

Please outline where this information can be found within the submission (e.g., sections or figure legends), or explain why this information doesn’t apply to your submission:

In this study, we performed genome sequencing from one individual of two non-model organism and comparative RNAseq between tissues using triplicate. In these studies, explicit power analysis was not discussed and no sample size estimates were made.

**Replicates**

- You should report how often each experiment was performed
- You should include a definition of biological versus technical replication
- The data obtained should be provided and sufficient information should be provided to indicate the number of independent biological and/or technical replicates
- If you encountered any outliers, you should describe how these were handled
- Criteria for exclusion/inclusion of data should be clearly stated
- High-throughput sequence data should be uploaded before submission, with a private link for reviewers provided (these are available from both GEO and ArrayExpress)

Please outline where this information can be found within the submission (e.g., sections or figure legends), or explain why this information doesn’t apply to your submission:

In the analysis of photosynthetic activity (Figure 2), we used three biological replicates of samples for each light conditions. In flow cytometry (Figure 4 – figure supplement 1), we performed three biological replicates of samples from different individuals and three times analyzed these same samples by FCM to obtain a technical replicate. In the tissue comparison RNA-Seq (Figure 5), three samples from different individuals were analyzed and assumed to be biological replicates. This information is described in the legends of each figure and in the corresponding text.

**Statistical reporting**

- Statistical analysis methods should be described and justified
- Raw data should be presented in figures whenever informative to do so (typically when N per group is less than 10)
- For each experiment, you should identify the statistical tests used, exact values of N, definitions of center, methods of multiple test correction, and dispersion and precision measures (e.g., mean, median, SD, SEM, confidence intervals; and, for the major substantive results, a measure of effect size (e.g., Pearson's r, Cohen's d)
- Report exact p-values wherever possible alongside the summary statistics and 95% confidence intervals. These should be reported for all key questions and not only when the p-value is less than 0.05.

Please outline where this information can be found within the submission (e.g., sections or figure legends), or explain why this information doesn’t apply to your submission:

The differences in photosynthetic activity were tested by Welch's two-sample t-test. The results are described in Figure 2, its legend and the corresponding text.

For tissue-to-tissue RNA-Seq, we statistically analyzed the expression variation using the false discovery rate, which is common in the field, and the GO-enrichment analysis was performed using Fisher's exact probability test. The results are shown in Figure 5, the legend and the corresponding text.

(For large datasets, or papers with a very large number of statistical tests, you may upload a single table file with tests, Ns, etc., with reference to sections in the manuscript.)

**Group allocation**

- Indicate how samples were allocated into experimental groups (in the case of clinical studies, please specify allocation to treatment method); if randomization was used, please also state if restricted randomization was applied
- Indicate if masking was used during group allocation, data collection and/or data analysis

Please outline where this information can be found within the submission (e.g., sections or figure legends), or explain why this information doesn’t apply to your submission:

The samples used in the experiments were randomly selected from the individuals collected and donated to the experiments. This is described in the method part.

**Additional data files (“source data”)**

- We encourage you to upload relevant additional data files, such as numerical data that are represented as a graph in a figure, or as a summary table
- Where provided, these should be in the most useful format, and they can be uploaded as “Source data” files linked to a main figure or table
- Include model definition files including the full list of parameters used
- Include code used for data analysis (e.g., R, MatLab)
- Avoid stating that data files are “available upon request”

Please indicate the figures or tables for which source data files have been provided:

All source data used in the analysis (Figure 1–6) were registered to the best of our ability. Some of the larger data are available on Figshare.
